# Peer Review of “Machine Learning for Risk Group Identification and User Data Collection in a Herpes Simplex Virus Patient Registry: Algorithm Development and Validation Study”

**DOI:** 10.2196/28919

**Published:** 2021-06-11

**Authors:** W Benjamin Nowell

**Affiliations:** 1 Global Healthy Living Foundation Nyack, NY United States

**Keywords:** data collection, herpes simplex, registry, machine learning, risk assessment, artificial intelligence, predictor, risk

*This is a peer-review report submitted for the paper “Machine Learning for Risk Group Identification and User Data Collection in a Herpes Simplex Virus Patient Registry: Algorithm Development and Validation Study.”*

## Round 1 Review

### General Comments

This paper [1] describes the process of designing and implementing an algorithm to lay the groundwork for developing a patient registry for people at risk of becoming infected with, or already living with, the herpes simplex virus (HSV). Specifically, the authors used a machine learning method (random forest modeling) to design an HSV patient registry that uses a limited number of lifestyle predictors for HSV infection and flare-up to choose the questions that are most relevant for registry participants. The authors were able to optimize the number of questions needed to achieve high accuracy in predicting HSV infection using this method. The authors situate their innovative method within the broader context of both the challenges associated with building a patient registry for a stigmatized condition, as well as the opportunities to create new registries by using publicly available data sets (eg, US National Health and Nutrition Examination Survey [NHANES]) and machine learning tools.

### Specific Comments

#### Major comments

1. The sections of the paper describing the method and results are strong, but sections such as the *Background*, *Challenges of Developing Patient Registries*, and *Discussion* need to be strengthened to set the context with a clearer focus. There are at least 3 examples of how this could be improved in my comments that follow.

2. First, a number of challenges associated with developing “a usable and effective patient registry” are highlighted, and the authors claim that their project “aimed to address these challenges by developing an innovative machine learning method for patient data collection and predictive analytics to improve data availability and quality in medical registries.” However, the approach and the 8-step process described by the authors would not actually address all of the four challenges equally well. The paper would be strengthened by being more specific and focused on which of the challenges are truly addressed by the process described here and which would likely require additional strategies. For example, it was not clear to me how this process would address patients’ or users’ concerns about privacy and control over their own data (the last of the 4 bullets) or necessarily meet the needs of patients. It is fine not to be able to address all of the challenges with the process you described, but I suggest you specify which of the challenges or concerns would be most directly solved by your process.

3. Second, you mention ArthritisPower, a research registry collaboration that brings together a patient advocacy organization and an academic medical center, in an effort to respond to the needs of patients with a smartphone app that facilitates symptom tracking, but the genesis of that registry/database is quite different than what you describe for the HSV registry. ArthritisPower participants already knew that they had a diagnosis of rheumatoid arthritis or another condition when they enrolled. Perhaps that is the point of citing the ArthritisPower example, since your process offers a contrast or alternative approach to assembling a patient registry, but this needs to be stated plainly if that is the case.

4. Third, you present an 8-step process that concludes with improvement of the precision of your model with real-world data. Later, in the *Discussion*, you note that future research and development of the system will go beyond that step to “examine important anonymity, consent, interoperability, and data security concerns, and develop and evaluate a holistic patient registry system (with a front-end user interface and a back-end data architecture).” These are important steps, but since these are outside the scope of this paper, I would take the opportunity earlier in the paper, like in the *Background* and registry challenges sections, to help narrow the focus to how your specific aims to build this model for HSV (and the 8-step process you outline) would be able to advance a registry’s efforts to the point where the steps beyond it might proceed with fewer barriers and more benefits to the end-users (patients and researchers).

5. The study appears to have been what one would consider secondary data analysis, but there was no mention of any human subjects or ethical research review in the United States or the United Kingdom. Typically, for this type of study in the United States, one might expect that the study was reviewed by an institutional review board (IRB) and received an exempt determination. As all of the authors are located in the United Kingdom, but the NHANES database is US-based, I am not sure what the established procedure should have been. Please describe any interaction with IRBs or institutional research ethics committees related to this research.

#### Minor Comments

6. Pg 3, lines 3-4: “...the value of these registries can be severely limited by a lack of high-quality...” Is the word “data” missing here? Either add a noun or perhaps edit to “lack of quality” followed by a full stop.

7. Pg 3, line 7: “...would provide significant benefits for research and clinical care.” Provide a few brief examples here (or later in the *Discussion* section) about the specific value for research and care since the sentence that follows is fairly general and applies to all registries, not specifically to one for HSV. What pressing questions about HSV could we answer with such a registry? How might this improve clinical care for HSV? This helps the reader understand why an HSV registry should be prioritized (ie, over other diseases) in the first place. This could be introduced in the *Background* and more fully explained in the *Discussion*.

8. Pg 6: Some explanation of the rationale for each of these criteria would be helpful; also, some of the criteria seem to overlap. For example, an extensive list of variables would naturally lend itself to the existence of a large number of rows, so I wondered why this needed to be stated in the second bullet. Perhaps the second requirement could be shortened to “clinically verified HSV diagnostic data.”

9. Pg 6, line 20: “…building a lifestyle-focussed questionnaire” only needs one “s” in “focused” for US readers. In fact, there are a handful of places throughout the paper where British vs American English conventions are inconsistent. For example, both spellings, “analysing” and “analyzing” were present. I am not sure what the exact editorial guidelines are for JMIR but would make sure this is consistent either way.

10. Pg 7, lines 16-17: “…confirmed negative or positive cases reported in NHANES were divided into two sub-datasets for training and validation of the model with a ratio of 0.8 to 0.2. The training dataset was used to train the model and the validation dataset was used for accuracy scoring.” Can you provide a rationale for dividing by the 0.8 to 0.2 ratio or at least give a citation for why this particular ratio was used? Is it due to statistical convention or because it maximizes the amount of data in the training data set with enough remaining data to conduct the validation or is there another reason?

11. Pg 8: “The model was designed to process the data in the following way...” The list of 8 steps in the process should perhaps be followed by a final sentence in that section to specify that the process or results described in this manuscript include steps 1 to 7 and that the next step (outside the scope of this paper) will be to conduct step 8 (ie, improve its precision with real-world data). This is implicit here, and then later mentioned more explicitly in the *Discussion*, but there it is accompanied by a longer list of next steps.

12. Pg 9, lines 24-26: “The model selected a set of 62 questions that form shorter sequences for each user based on their age and gender. On average, a user would be asked 40 questions, with a minimum of 21.” Does this mean the max set of questions a person might answer is 62? If so, state that explicitly. If not, please clarify. Currently, it reads as though you have provided an average number of questions that participants must answer, and a minimum number, but no maximum. If known, it might also be informative to readers to state the average amount of time it took participants to answer the original ~150 NHANES questions versus the 40 questions. This gives the reader a more concrete sense of how much you were able to reduce participant burden with your optimized questionnaire for HSV compared to the original. Even an estimate of the time, if the exact time is not known, would be instructive.

13. Pg 10, line 1: The header would be clearer if it read, *NHANES Questions With Added Questions* or *NHANES Questions With Supplementary Questions*. The word “added” seems to be dangling.

14. Pg 11, lines 13-14: “The ultimate aim of this project is to increase the quality and quantity of data collected and improve the probability of users disclosing sensitive information and volunteering for clinical trials.” It is intuitive to assume so, but is there evidence you can cite that supports the fact that fewer questions are better for more sensitive information, above and beyond the usual benefit of minimizing participant burden for any questionnaire, and is there evidence that this ultimately leads to patients providing more data? I would suppose it can if the optimized original survey frees you up to then ask other questions.

15. Pg 11, line 21: Consider replacing “on” with “regarding” (ie, “to generate more insights regarding what questions…” instead of “to generate more insights on what questions…”).

16. Pg 12, lines 5-6: “…and members of the public.” Please specify what is meant by the eligible “public” users of the platform. There are presumably some differences in the data that would be available to users in different places or different nomenclature based on different health care systems or linguistics and terms in different places. Is this for US users, UK users, or both?

17. Pg 12, line 22: What is meant by “pseudo-anonymised data”? Please be specific or provide an example.

## Round 2 Review

### General Comments

The authors have addressed the suggestions and comments from the first review, and I believe this paper is ready for publication, pending minor revisions.

### Specific Comments

#### Major Comments

Insufficient edits were made to address the concerns I had about clarity and consistency of syntax in the first review. Please address syntax and copyediting issues throughout. I have specified several suggested edits in the *Minor Comments*, but this paper needs a thorough copyediting review by the authors or a paid service. Moreover, I saw that there are still many places where British English is used rather than American English. This should be a relatively easy change to make via MS Word. For example, “analyz-” should be used in lieu of “analys-” (except in the case of the word “analysis”), “maximise” should be “maximize,” “optimize” should be “optimize,” and in the US context, we use “-er” in lieu of “-re” (eg, patient-centered).

#### Minor Comments

1. This sentence is confusing; it needs to be made more concise or broken into two sentences instead of one:

*Actual*: For example, the lack of data on people who are living with HSV but not developed symptoms calls to specific need to collect data outside of clinical settings from populations who have not developed symptoms and are not motivated to complete extensive data collection forms, therefore requiring non-intrusive and time-efficient methods to reliably identify high-risk groups.

*Suggested*: For example, the lack of data on people who are living with HSV but have not developed symptoms requires collecting data outside of clinical settings from populations who may not be motivated to complete extensive questionnaires or, worse, take offense at being asked to do so. Therefore, nonintrusive and time-efficient methods are necessary to reliably identify high-risk groups.

2. *Actual*: One type of decision support model, decision trees, can be applied to analyse the flows of user-generated content and to determine the strategy that is the most efficient and the most likely to be successful means of achieving a certain goal.

*Suggested*: One type of decision support model, decision trees, can be applied to analyze the flows of user-generated content, and to determine the strategy that is most efficient and most likely to successfully achieve a certain goal.

3. *Actual*: The ArthritisPower registry platform also proved a more effective means of engaging patients with research and enabling patient-generated data capture, however is limited to users who had been already diagnosed and are actively motivated to participate. In addition to increased patient engagement with research, the growing focus on patient-centred care has resulted in an increased place for patient reported outcomes in clinical care and research, and are a key component of patient registries.

*Suggested*: The ArthritisPower registry platform has proved to be an effective means of engaging patients to participate in research and enabling patient-generated data capture; however, the registry is limited to users who have already received a physician diagnosis and are actively motivated to participate. In addition to increased patient engagement with research, the growing focus on patient-centered care has led to a new emphasis on the use of patient-reported outcome measures in clinical care and research, and PROs now constitute a key component of patient registries.

4. Each of the four challenges listed in the *Introduction* should begin with a shorthand label of the challenge to make it easier to read. For example:

1. Efficient use of data. Collecting sufficient and high-quality data...
2. Patient-centric design. To be usable and effective...
3. Selection bias...
4. Privacy concerns...

5. *Actual*: Similarly, the patient-centric design (the second challenge) requires the consideration of user expectations such as ease of completion, avoiding, where possible, a significant effort, both mental and physical, which also can contribute to improve the selection bias (challenge three), by, for example increasing completion rates by those less motivated or having less capacity.

*Suggested*: Similarly, a patient-centric design (challenge 2) requires consideration of the user experience, which includes minimizing participant burden. This may also ultimately reduce selection bias (challenge 3) by increasing completion rates.

6. *Actual*: Therefore, this project is aimed primarily at addressing the challenges of long, time-consuming questionnaires with many sensitive questions for creating a prediction model that would reliably assess whether a particular person has an increased risk of HSV. We have explored the applications of innovative machine learning methods for optimizing the question list while maintaining high quality and relevance of the collected data.

*Suggested*: Therefore, this project is aimed primarily at addressing the challenges associated with time-consuming questionnaires containing sensitive questions by creating a prediction model to reliably assess whether a particular person has an increased risk of HSV. We explored the applications of innovative machine learning methods in order to optimize the questions asked of participants, while maintaining the high quality and relevance of collected data.

7. *Actual*: For the future studies, it is suggested to integrate this approach with privacy-preserving and trust-enabling solutions to strengthen all four of the areas.

*Suggested*: For future studies, we suggest integrating this approach with privacy-preserving and trust-enabling solutions to more comprehensively address the four challenges described above.

8. *Actual*: In the current study we design and test an algorithm that follows steps 1-7. The step 8, the improvement of precision as the result of integration within the live data collection system, is intended as a direction for the future work.

*Suggested*: In this study, we designed and tested an algorithm that follows steps 1 to 7. Step 8, improvement of precision via integration with a live data collection system, is intended as a direction for future work.

9. *Actual*: Researchers will be able to use the registry to complement clinical research and facilitate patient recruitment for clinical trials. Researchers will need to register, be verified by the system administrator, and login to their account before accessing pseudo-anonymized data, that is the data that underwent procedures to remove personally identifiable information and is anonymized, where however the links to the original personal data are preserved.

*Suggested*: Researchers will be able to use the registry to complement clinical research and facilitate patient recruitment for clinical trials. Researchers will need to register, be verified by the system administrator, and login to their account before accessing pseudo-anonymized data (ie, data where personally identifiable information have been removed, but links to the original personal data are preserved).
